# Composite A_2_M_6_O_13_ anodes (A = Li, Na; M = Ti, Zr) for Li–Na dual cation batteries: a theoretical investigation

**DOI:** 10.1039/d5ra10064j

**Published:** 2026-03-02

**Authors:** Duc Toan Truong, Yohandys A. Zulueta, My Phuong Pham-Ho, An-Giang Nguyen, Chi M. Phan, Minh Tho Nguyen

**Affiliations:** a Laboratory for Chemical Computation and Modeling, Institute for Computational Science and Artificial Intelligence, Van Lang University Ho Chi Minh City Vietnam; b Faculty of Applied Technology, Van Lang School of Technology, Van Lang University Ho Chi Minh City Vietnam; c Departamento de Física, Facultad de Ciencias Naturales y Exactas, Universidad de Oriente Santiago de Cuba CP 90500 Cuba yzulueta@uo.edu.cu; d Faculty of Chemical Engineering, Ho Chi Minh City University of Technology (HCMUT) 268 Ly Thuong Kiet Street Ho Chi Minh City Vietnam; e Vietnam National University Ho Chi Minh City Linh Trung, Thu Duc Ho Chi Minh City Vietnam; f Center for Environmental Intelligence, VinUniversity Hanoi 100000 Vietnam tho.nm@vinuni.edu.vn; g College of Engineering and Computer Science, VinUniversity Hanoi 100000 Vietnam; h Discipline of Chemical Engineering, WASM, Curtin University Perth 6045 Australia

## Abstract

The development of advanced anode materials is critical for improving the efficiency and durability of alkali-ion batteries. In this study, large-scale molecular dynamics simulations are employed to investigate the transport properties of A_2_M_6_O_13_ (A = Li, Na; M = Ti, Zr) compounds in mono-, bi-crystalline and composite forms. Grain boundaries exert a decisive influence on ion migration in enhancing Na^+^ mobility in bi-Na_2_Zr_6_O_13_ but slightly restrict transport in bi-Na_2_Ti_6_O_13_. Composite architectures integrating both Li- and Na-based phases (Li_2_Zr_6_O_13_@Na_2_Ti_6_O_13_, LZNTO; Li_2_Ti_6_O_13_@Na_2_Zr_6_O_13_, LTNZO) exhibit superior conductivity compared to Na-only counterparts, underscoring the higher intrinsic mobility of Li^+^ ions. Population-weighted mean square displacement analysis confirms that effective diffusivity and conductivity in dual-cation composites are mathematically equivalent to the sum of species-resolved contributions, thereby capturing simultaneous transport effects. Of the studied systems, Na_2_Ti_6_O_13_ demonstrates excellent Na^+^ transport with the lowest activation energy, while Li-containing composites achieve moderate conductivity through synergistic Li^+^/Na^+^ migration. These findings provide evidence of synchronized transport in dual-cation titanate/zirconate composites, establishing LZNTO and LTNZO as promising anode candidates for next generation Li–Na dual-cation battery systems.

## Abstract 2

The development of advanced anode materials is critical for improving the efficiency and durability of alkali-ion batteries.

## Graphical abstract

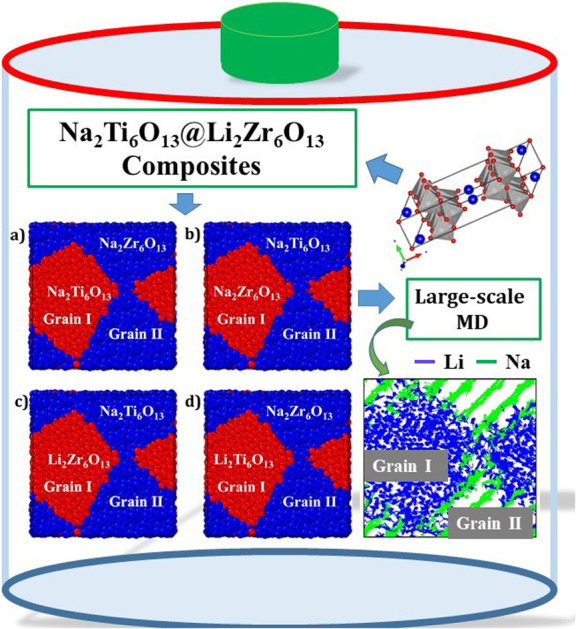

## 1 Introduction

Electrode materials are the fundamental components that govern both the energy storage and release in alkali-ion batteries, as they host the reversible insertion and extraction of alkali ions.^1–6^ The anode, typically operating at lower potentials, serves as the reservoir for Li^+^ or Na^+^ during charging, and its structural stability, ion mobility, and interfacial chemistry dictate the achievable capacity, rate performance and cycle life.^1–3^ In contrast, the cathode functions as the source and host of alkali ions at higher potentials, with its redox activity and framework robustness largely determining the operating voltage, energy density, and long-term durability of the cell.^1–9^ Together, the interplay between anode and cathode defines the thermodynamic window, transport kinetics, and overall performance of alkali-ion batteries.^1–9^

Lithium hexatitanate (Li_2_Ti_6_O_13_) has been recognized as a viable negative electrode material for lithium-ion batteries (LIBs), in part owing to its tunnel structure and large surface area. It possesses an open-cell voltage in the range of 1.5–1.7 V, a theoretical capacity of 170 mAh g^−1^, and a dc-conductivity of 5.6 × 10^−6^ S cm^−1^ at 25 °C.^7–11^ These electrochemical and structural attributes make Li_2_Ti_6_O_13_ a compelling candidate for anode applications.^7–11^ However, despite such intrinsic properties, extensive experimental strategies have been pursued to enhance its performance, with findings indicating that lithium pentatitanate exhibits even greater promise as an anode material.^7–13^

On the other hand, sodium hexatitanate (Na_2_Ti_6_O_13_) has been considered as a candidate for sodium-ion battery (SIB) anode, equally due to its tunnel-like crystal structure and large surface area which facilitate sodium-ion diffusion. It is characterized by an open-cell voltage of approximately 0.8–1.2 V, a theoretical capacity of around 140 mAh g^−1^, and a moderate ionic conductivity that supports stable charge–discharge cycling.^14–16^ These electrochemical and structural features position Na_2_Ti_6_O_13_ as a viable anode material for SIBs. However, various experiments that have been explored to optimize its electrochemical properties suggested that other sodium titanates, such as Na_2_Ti_3_O_7_, may offer superior Na-ion storage performance under certain conditions.^15–17^

Previous computational studies exploring the anode performance of lithium hexazirconate (Li_2_Zr_6_O_13_) revealed that this Zr compound shares the lattice structure of Li_2_Ti_6_O_13_ and more importantly maintains similar electronic and mechanical characteristics.^11,13^ Its open-cell voltage aligns closely with that of Li_2_Ti_6_O_13_ and other comparable anode materials.^7–11,13^ Numerous experimental approaches were dedicated to optimization of structurally related materials.^7–20^ For instance, Li_2_Ti_6_O_13_ was integrated as a co-material to stabilize the structure and improve the ionic conductivity in the Li_4_Ti_5_O_12_–Li_2_TiO_3_ composite, surpassing the conductivity of Li_4_Ti_5_O_12_.^21,22^

Beyond titanates and zirconates, other oxide and composite systems have also been explored to enhance ionic conductivity and cycling stability.^18–20^ For instance, carbon-coating-free β-Li_2_TiO_3_ was characterized with a specific capacity of 200 mAh g^−1^ over 100 cycles, retaining 170 mAh g^−1^ after 500 cycles and achieving a coulombic efficiency exceeding 97%.^18^ Graphene-supported Li_2_SiO_3_@Li_2_SnO_3_ composite, synthesized *via* hydrothermal methods, presents an initial specific capacity of 1016.5 mAh g^−1^, with a sustained performance at 440.8 mAh g^−1^ after 200 cycles, a result attributed to the synergistic interaction between composite components.^19^ In a separate study, impedance measurements explored the temperature-dependent electrical properties of biphasic sodium titanate/poly-*o*-methoxyaniline (Na_2_Ti_3_O_7_/Na_2_Ti_6_O_13_/POMA) composites across different POMA concentrations, revealed some distinct electrical behaviour as compared to individual materials.^20^

Atomistic simulations serve as a powerful means for establishing structure–property relationships and thereby facilitating theoretical design of novel materials.^23–25^ Classical MD computations provide us with crucial insights into transport properties within large systems.^23–25^ Previous studies on Li_2_Ti_6_O_13_ addressed its thermodynamic stability, lattice characteristics, doping effects, and partial diffusion processes.^11,13,24–28^ To our knowledge, comprehensive MD simulations analysing the transport properties of Na_2_Ti_6_O_13_ are not reported yet.

In a recent work, we conducted large-scale MD simulations to investigate transport properties of Li_2_Ti_6_O_13_ and Li_2_Zr_6_O_13_ in both mono-crystalline and bi-crystalline forms, as well as their composite structure, Li_2_Ti_6_O_13_@Li_2_Zr_6_O_13_.^29^ While both monocrystalline and bi-crystalline Li_2_Zr_6_O_13_ possess comparable transport behaviour, the composite materials which integrate both compounds, result in superior diffusion coefficients and dc-conductivity. This enhancement was attributed to the lithium interstitial mechanism and influence of grain boundaries that facilitate ion transport.^29^

Dual cation systems (DCS) in which both Li^+^ and Na^+^ ions participate in the charge–discharge process, have recently attracted attention as a strategy to expand the design space of alkali-ion systems.^30–36^ Incorporation of two mobile species within a single anode framework enables simultaneous transport mechanisms, where the intrinsically higher mobility of Li^+^ balances the abundance and distinct insertion potential of Na^+^.^30–36^ Tunnel-structured titanates and zirconates are particularly suitable hosts as their open frameworks can accommodate simultaneous migration of both cations while maintaining structural stability.^24–29^ Computational and experimental studies demonstrated that composite architectures combining Li- and Na-based phases facilitate interstitial ion exchange and grain-boundary-assisted diffusion, thereby enhancing ionic conductivity and mitigating degradation associated with single-cation cycling.^14–17,29^ These findings establish dual cation anode as a promising frontier for next generation alkali ion batteries, offering improved kinetics, durability, and versatility as compared to conventional single-cation electrodes.^30–36^

Beyond single-cation systems, composite structures that integrate both Li_2_Ti_6_O_13_ and Na_2_Zr_6_O_13_ or Li_2_Zr_6_O_13_ and Na_2_Ti_6_O_13_ offer a unique opportunity to exploit dual cation transport. The coexistence of both Li^+^ and Na^+^ cations within tunnel-type frameworks enables cooperative migration pathways, where the smaller Li^+^ ions facilitate interconnected diffusion channels whereas the larger Na^+^ ions contribute to structural stabilization and broaden the accessible voltage window. Such dual cation composites therefore represent a strategic design frontier, combining the intrinsic mobility of Li^+^ with the abundance and cost effectiveness of Na^+^ to achieve enhanced conductivity and durability as compared to conventional single cation electrodes.^30–36^

Grain boundaries exert a profound influence on transport behaviour in polycrystalline materials.^37–42^ In this context, we conduct large-scale MD computations to elucidate the transport properties of both mono- and bi-crystalline Na_2_B_6_O_13_ (B = Ti, Zr). Furthermore, we explore novel ceramic composite materials, making the first investigation on the composite of both Na_2_Zr_6_O_13_ and Na_2_Ti_6_O_13_ compounds, including also a mixture of both sodium and lithium hexatitanate/hexazirconate materials. Our analysis specifically assesses the impact of grain boundaries on transport properties, providing insights that can bring in future experimental advancements.

## 2 Computational details

Optimization of ion transport properties aims to ensure a rapid alkali ion transport which is essential for achieving stable cycling performance and minimizing undesirable reactivity between the anode and the solid-state electrolyte.^37–42^ In this study, we employ the large-scale atomic-molecular massively parallel simulator (LAMMPS) program to perform molecular dynamics (MD) simulations with periodic boundary conditions to analyse the alkali-ion transport mechanism.^43^ Force-field parameters used in these simulations are sourced from previous reports,^27,29,44^ where the Buckingham potential approximation is applied for short-range interactions, and coulombic interactions describe long-range electrostatic forces. To maintain consistency in computational methodology and for the sake of meaningful comparison, we apply the same MD conditions outlined in our previous studies.^27,44^

**Fig. 1:**
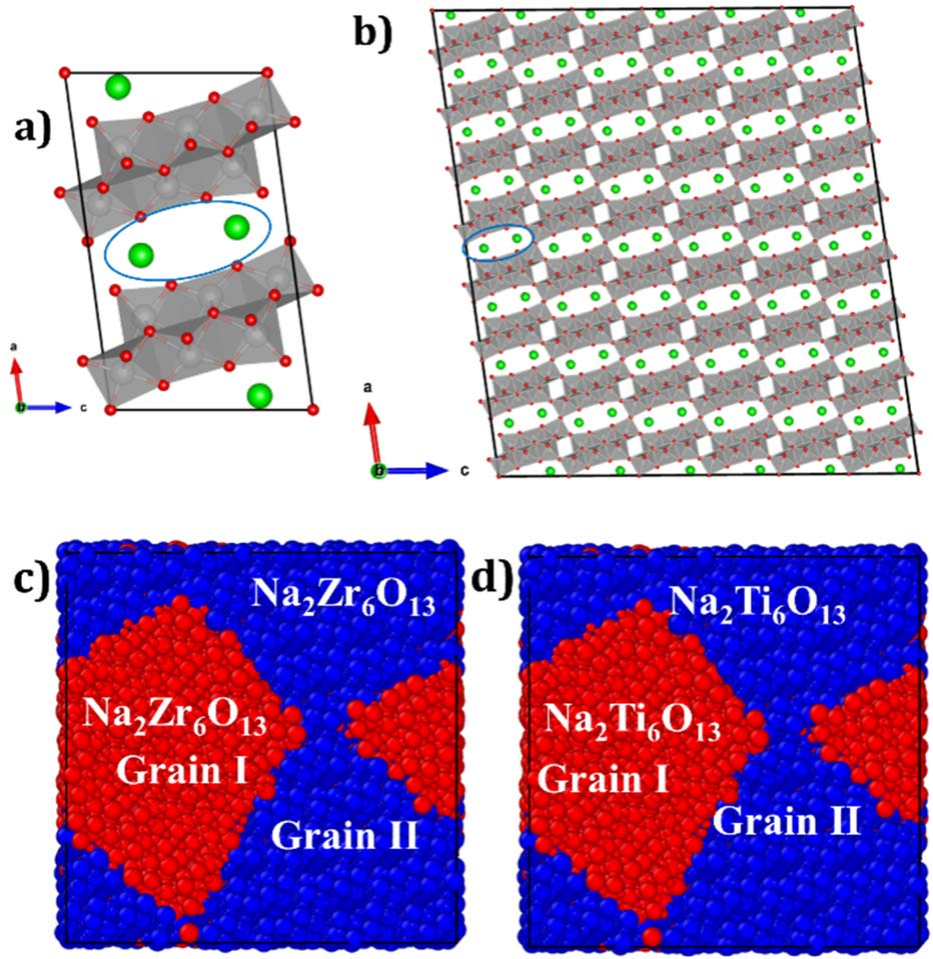
Crystal structure of Na_2_B_6_O_13_ (B = Ti^4+^ or Zr^4+^): (a) unit cell highlighting [NaO_4_] channels, Na^+^ (green), O^2−^ (red), and [BO_6_] octahedra (light brown); (b) monocrystalline view. Bi-crystalline samples: (c) Na_2_Zr_6_O_13_ (bi-NZO) and (d) Na_2_Ti_6_O_13_ (bi-NTO), with grains distinguished by colour.

The mono- and bi-crystalline structures of Na_2_B_6_O_13_ (B = Ti^4+^, Zr^4+^) are generated using the Voronoi tessellation method implemented in the Atomsk code.^36^ Monocrystalline Na_2_B_6_O_13_ samples consist of a single grain within a 4 × 15 × 6 supercell containing 360 unit cells shown in Fig. 1b. Conversely, bi-crystalline Na_2_Zr_6_O_13_ (bi-NZO) and Na_2_Ti_6_O_13_ (bi-NTO) samples include two randomly oriented grains, referred to as Grain I and Grain II, as displayed in Fig. 1c and d, respectively.

**Fig. 2:**
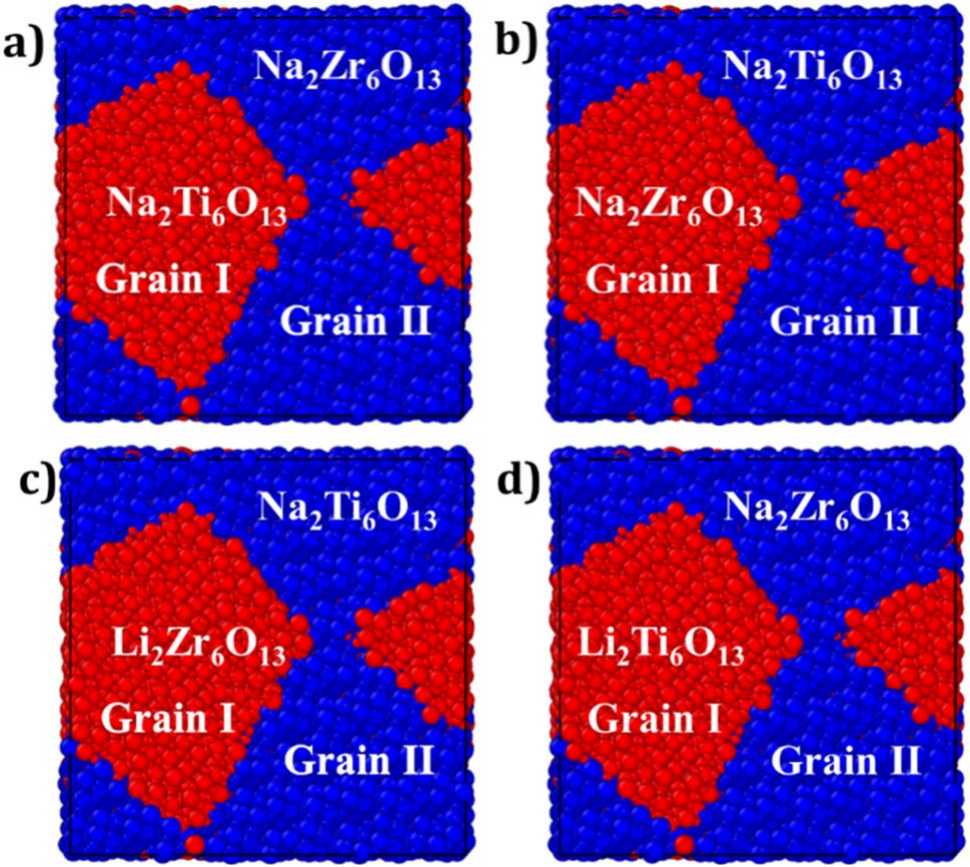
Mixed bi-crystalline samples: (a) Na_2_Ti_6_O_13_@Na_2_Zr_6_O_13_ (NTZO), (b) Na_2_Zr_6_O_13_@Na_2_Ti_6_O_13_ (NZTO), (c) Li_2_Zr_6_O_13_@Na_2_Ti_6_O_13_ (LZNTO) and (d) Li_2_Ti_6_O_13_@Na_2_Zr_6_O_13_ (LTNZO). Colour coding refers to a different grain.

Fig. 2 illustrates the mixed bi-crystalline composites. The composite structures, including Na_2_Ti_6_O_13_@Na_2_Zr_6_O_13_ (labelled NTZO) Na_2_Zr_6_O_13_@Na_2_Ti_6_O_13_ (labelled NZTO), Li_2_Zr_6_O_13_@Na_2_Ti_6_O_13_ (LZNTO) and Li_2_Ti_6_O_13_@Na_2_Zr_6_O_13_ (LTNZO), are generated from bi-crystalline simulation boxes. In these composites, the notation (Grain I)@(Grain II) denotes the structural composition: in NTZO, Grain I consists of Na_2_Ti_6_O_13_, while Grain II is composed of Na_2_Zr_6_O_13_; in NZTO, Grain I contains Na_2_Zr_6_O_13_, while Grain II corresponds to Na_2_Ti_6_O_13_. Similarly, for LZNTO, Grain I is composed of Li_2_Zr_6_O_13_, whereas Grain II contains only Na_2_Ti_6_O_13_.

As in our previous work,^29^ “composition” denotes the A_2_B_6_O_13_ (A = Li^+^, Na^+^, B = Ti^4+^, Zr^4+^) phase present in each grain. Bi-crystalline/composite cells use 60 × 60 × 60 Å^3^ boxes; monocrystalline cells use 4 × 15 × 6 unit-cell supercells (61.71 × 56.63 × 55.94 Å^3^), thus ensuring comparable system sizes.

The A_2_O Schottky defect type is typically observed in these structures because of its relatively low energetic cost.^13,27^ Under the A_2_O Schottky scheme, for each O^2−^ vacancy two alkali vacancies are required for charge neutrality.^27^ This defect scheme leads to increase the alkali vacancies with direct implication on the alkali transport properties. In this sense, to investigate alkali-ion migration, a low concentration of 0.09 of A^+^ ion vacancies, compensated by O^2−^ anion vacancies (as per the A_2_O Schottky defect mechanism), is introduced into the simulation boxes.

During the tessellation process, certain ions may be positioned excessively close to one another or even superimposed. To resolve this issue, these ions are either removed or separated before defect incorporation into bi-crystalline samples. Any excess charge resulting from defect introduction is neutralized by compensatory A^+^ vacancies. The quantity of ions and defect concentration are given in the SI file (Table S1).

To ensure the reliability of simulations, each system undergoes equilibration using an isothermal-isobaric ensemble (NPT). After stabilization, production simulations are carried out using a constant volume-temperature (NVT) ensemble where the mean square displacement (MSD) of Li^+^ and Na^+^ ions is recorded to determine the diffusion coefficient (*D*).

The diffusion coefficient is determined from the slope of the mean square displacement (MSD) plots using eqn (1):

1 MSD = 6*Dt*

where *t* represents the simulation time. In the molecular dynamics simulations conducted for this study, a production run of 2 ns is employed with a time step of 2 fs, and the temperature is varied within the range of 900–1400 K.

To ensure reproducibility, LAMMPS input files for monocrystalline and bi-crystalline Na_2_B_6_O_13_, as well as composite structures are provided in the SI file, offering additional simulation details.

The computed diffusion data are subsequently converted into dc-conductivity using the Nernst–Einstein eqn (2):

2 *σ*(*T*) = *H*_V_*Nq*^2^*D*(*T*)/*k*_B_*T*

where *σ*(*T*) and *D*(*T*) denote the dc-conductivity and diffusion coefficient at temperature (*T*), respectively; *N* represents the charge density of the mobile ion, *q* its charge, *k*_B_ the Boltzmann constant, and *H*_V_ the Haven's ratio which accounts for the influence of the external electric field on charge carrier mobility in real materials.^23,25,39,44^ Due to the lack of experimental measurements of diffusion and conduction processes for Na_2_Ti_6_O_13_, Li_2_Ti_6_O_13_ and the new Na_2_Zr_6_O_13_–Li_2_Zr_6_O_13_ structures, the value *H*_V_ = 1 is assumed. This assumption for *H*_V_ is expected to induce some deviations in calculated predictions.

Both diffusion and conduction mechanisms follow thermally activated kinetics that adhere to the Arrhenius-type eqn (3):

3 *Δ*(*T*) = *Δ*_0_ exp(−*E*^Δ^_a_/*k*_B_*T*)

where *Δ*_0_ represents the pre-exponential factor (*Δ*(*T*) → *Δ*_0_, *T* → *∞*), and *E*^Δ^_a_ the activation energy. The Arrhenius dependence is evaluated for conduction (*Δ* = *σ*) as well as for diffusion (*Δ* = *D*). Alongside activation energy, both diffusion and dc-conductivity values at 25 °C serve as key parameters characterizing the transport properties of ionic materials.^23,25,39,44^

For the dual cation composites (LZNTO and LTNZO), the mean square displacement (MSD) at each temperature is evaluated using a population-weighted definition eqn (4) that accounts for both Li^+^ and Na^+^ migrations:4
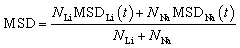


The effective diffusivity (*D*_eff_) is then obtained following eqn (1), while the conductivity is derived from eqn (2), where *N* represents the total charge density of mobile ions. The activation energy is subsequently determined from the Arrhenius dependence given in eqn (3). This formulation yields effective diffusivity and conductivity (*σ*_eff_) values that are mathematically equivalent to the sum of the species-resolved contributions. By reporting both species-specific and combined transport metrics, we explore not only the intrinsic mobility of each cation, but also the simultaneous effects that emerge in dual cation framework.

## 3 Results and discussion

### 3.1 Tracking Na^+^-ion transport in mono-, bi-crystalline Na_2_B_6_O_13_ with B = Ti and Zr samples

**Fig. 3:**
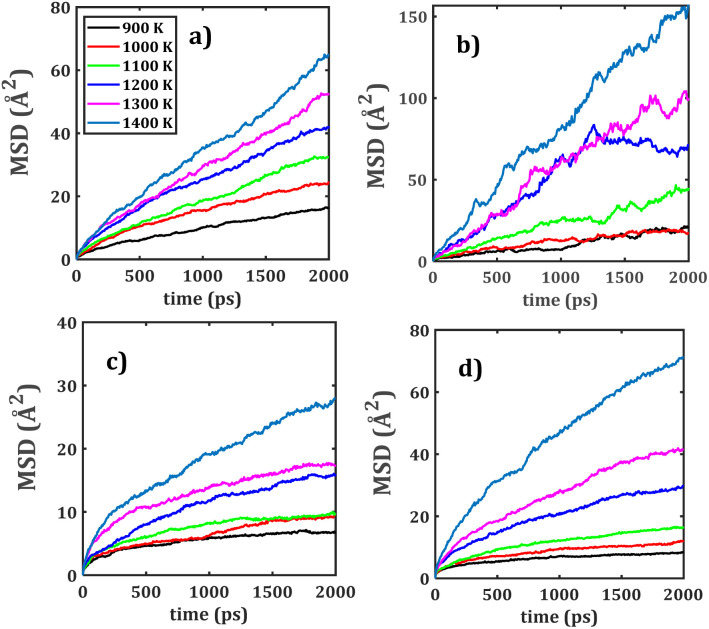
MSD *versus* simulation time of monocrystalline (a) Na_2_Ti_6_O_13_ (NTO), (b) Na_2_Zr_6_O_13_ (NZO), bi-crystalline (c) Na_2_Ti_6_O_13_ (bi-NTO) and (d) Na_2_Zr_6_O_13_ (bi-NZO).

Fig. 3a and b present the temporal evolution of mean square displacement for monocrystalline Na_2_Ti_6_O_13_ and Na_2_Zr_6_O_13_ samples as a function of temperature. Notably, the MSD plots (*i.e.* the range) for Na_2_Zr_6_O_13_ consistently appear to be higher than those for Na_2_Ti_6_O_13_ at higher temperature, indicating an enhanced Na^+^ ion mobility in Na_2_Zr_6_O_13._ However, Fig. 3b suggests that the monotonic increase of the slope in monocrystalline Na_2_Zr_6_O_13_ is affected.

In NZO the MSD rises until ∼1200 ps and then plateaus, suggesting saturation of accessible migration pathways. This likely reflects full utilization of Na^+^ vacancies and a transition to reversible hopping within the monocrystalline lattice. The diffusion coefficient, determined using eqn (1), is significantly larger in monocrystalline NZO as compared to NTO. The higher Na^+^ mobility in NZO sytems from the larger Zr^4+^ ionic radius (0.115 Å),^38^ which widens conduction channels and increases Na–Na jump lengths by expanding lattice and octahedral dimensions.^26,45^

In NZO, the larger Zr–O bond lengths result in wider conduction channels, reduced steric hindrance, and potentially more accessible intermediate configurations along the migration pathway.^24,46,47^ Conversely, the more compact Ti-based framework in NTO yields narrower tunnels, higher lattice rigidity, and a more constrained migration landscape.^24,26^ Thus, even a modest difference in cation size can propagate through the lattice architecture, shaping both the static and dynamic transport properties of these tunnel-type structures. Since NZO exhibits a longer Na–Na distance (3.908 Å) than its isostructural NTO counterpart (3.737 Å),^24,26^ the increased jump length directly enhances Na^+^ mobility, and higher diffusion coefficients are therefore anticipated for monocrystalline Na_2_Zr_6_O_13_.

Fig. 3c and d illustrate the MSD evolutions for both bi-crystalline Na_2_Ti_6_O_13_ and Na_2_Zr_6_O_13_ samples. Bi-crystalline samples show a more linear MSD *versus* time, consistent with grain boundaries creating extended disorder and additional vacancy–rich pathways that alter Na^+^ migration. Similar to monocrystalline systems, bi-NZO demonstrates superior diffusion characteristics-at higher temperatures-as compared to its Ti^4+^ counterpart. Combined effects of the [BO_6_] octahedral distortion and grain boundary interactions thus affect transport properties in bi-crystalline samples relative to mono-crystalline counterparts.^11,37,38^

**Fig. 4:**
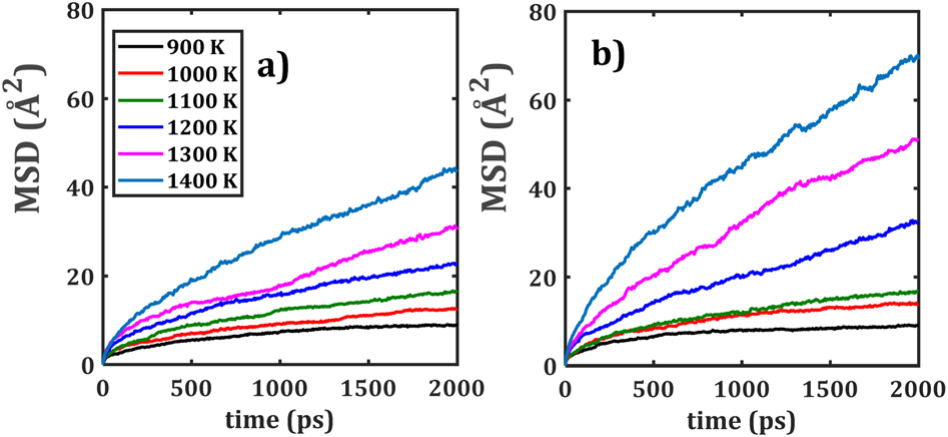
MSD *versus* simulation time of mixed bi-crystalline samples: (a) NTZO and (b) NZTO.

Fig. 4 shows the MSD evolution for both mixed bi-crystalline samples. In analogy with pristine bi-crystalline samples, the presence of grain boundaries affects the long-range Na^+^ migration across the simulation box by providing more accessible Na^+^ sites (*i.e.*, Na^+^ vacancies) within the material.

**Fig. 5:**
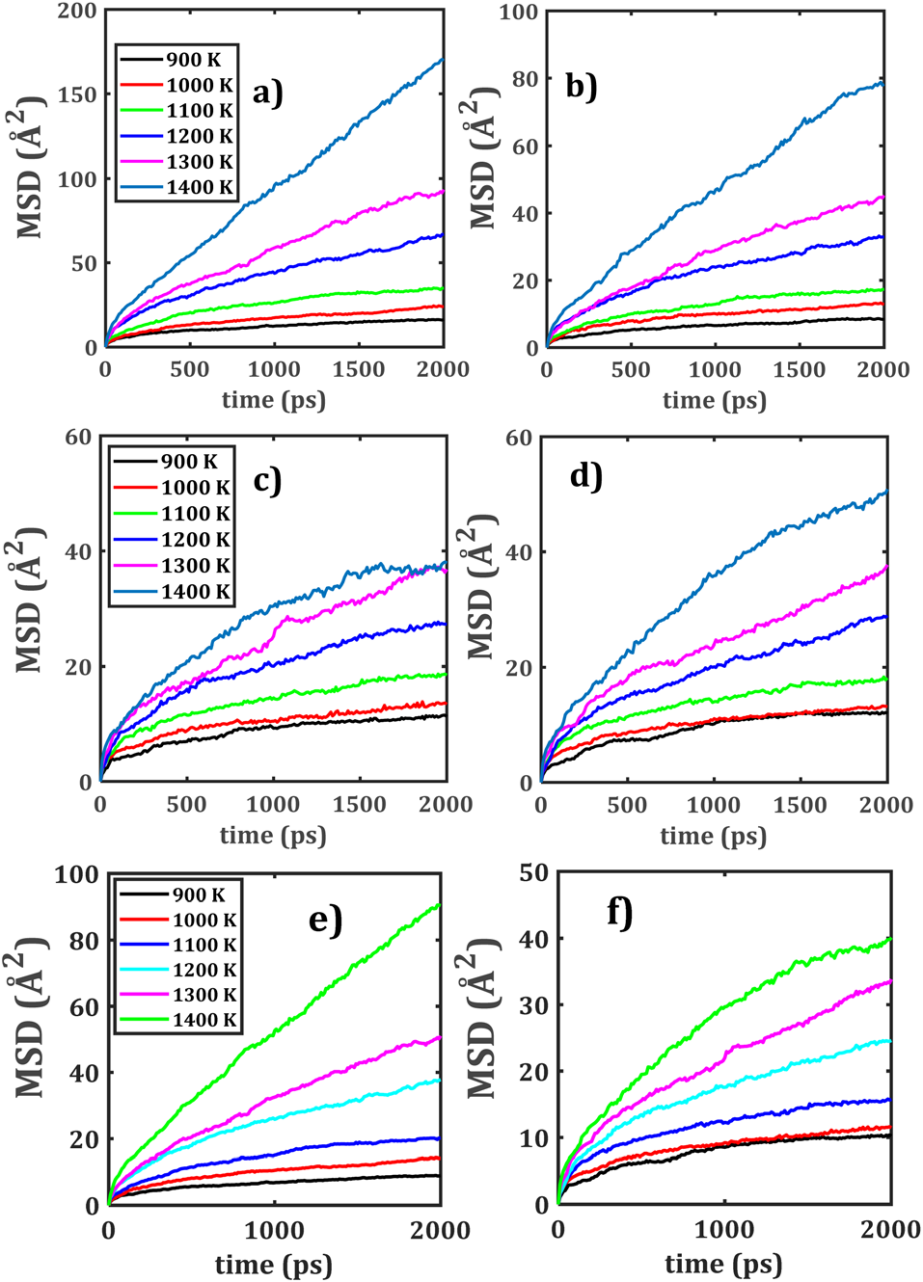
MSD plots for mixed bi-crystalline LTNZO and LZNTO composites. Panels (a) and (c) depict Li^+^ migration, panels (b) and (d) show Na^+^ migration, while panels (e) and (f) Li^+^/Na^+^ population-weighted MSD in LTNZO and LZNTO, respectively.

Fig. 5a displays the Li^+^ migration in bi-crystalline LTNZO, whereas Fig. 5b depicts the Na^+^ migration in the same material. The primary distinction between these figures lies in the transport dynamics of each ion. Due to its smaller ionic radius, Li^+^ follows highly interconnected migration pathways, thus facilitating extended Li^+^ migration throughout the lattice and enabling long-range diffusion across the material. In contrast, Na^+^ migration is more localized, because its larger size imposes higher energy barrier restricting its transport. However, grain boundaries affect Na^+^ mobility due to its resistance.^11,37,38^

Fig. 5c represents Li^+^ migration in bi-crystalline LZNTO, while Fig. 5d illustrates Na^+^ migration in the same material. Similar to its behavior in LTNZO, Li^+^ in LZNTO maintains a high mobility in which grain boundaries modifies the diffusion pathways. Na^+^ migration in LZNTO follows a comparable trend to LTNZO where grain boundaries actually aid ionic transport, but diffusion remains comparatively constrained due to larger Na^+^ size.^38^ The presence of Zr^4+^ in LZNTO can subtly influence migration kinetics. When comparing LTNZO to LZNTO, the Li^+^ incorporation brings in superior transport properties, beneficial from interconnected migration channels. For its part, while Na^+^ migration is improved by grain boundaries, it remains inherently more restricted due to its size-dependent diffusion constraints.

The collective MSD (Fig. 5e and f) provide a direct evidence of the distinct yet complementary roles of Li^+^ and Na^+^ in dual cation composites. The Li^+^ ions exhibit consistently steeper MSD slopes, reflecting their smaller ionic radius. In contrast, Na^+^ ions display more localized hopping dynamics, with mobility strongly enhanced at grain boundaries where additional vacancies and distorted coordination environments may facilitate transport.

### 3.2 Transport properties in mono-, bi-crystalline and mixed bi-crystalline samples

To quantitatively assess the A^+^ transport properties of the compounds considered, the Arrhenius-type dependence of both diffusion and conduction processes is now analysed. Using the diffusion data collected for each sample, the dc-conductivity at various temperatures is determined using eqn (2).

**Fig. 6:**
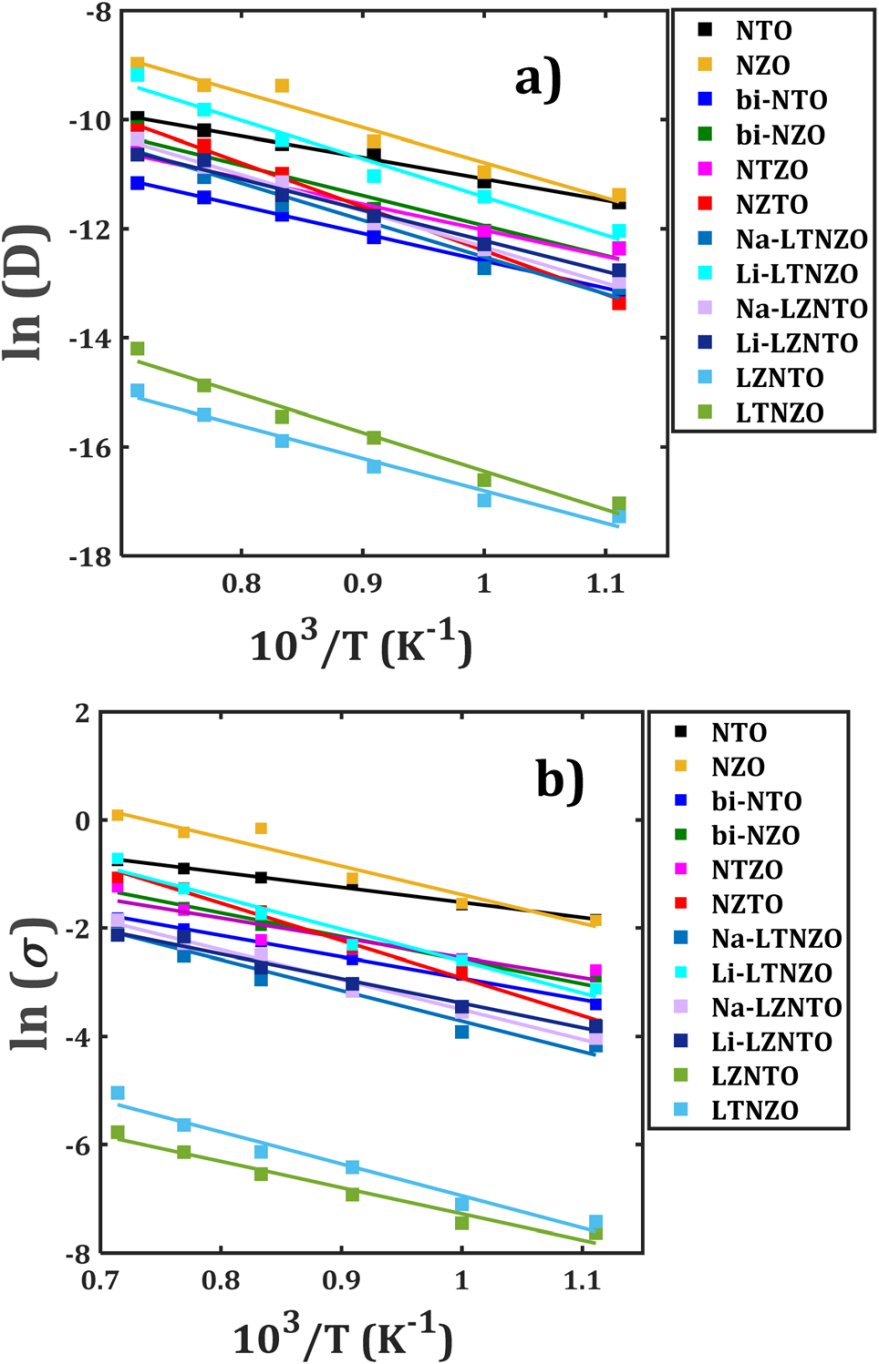
Arrhenius-type dependence of (a) diffusion coefficient, and (b) dc-conductivity with respect to temperature of mono-, bi- and mixed bi-crystalline samples. Lines represent the Arrhenius-type fit.

Fig. 6 illustrates the Arrhenius-type behaviour of the diffusion and conduction data of mono-, bi-crystalline and mixed bi-crystalline samples. The ionic transport properties of the studied materials are quantitatively assessed through Arrhenius fitting, allowing the determination of key parameters including the diffusion activation energy (*E*_a_^*D*^), diffusion coefficient at 25 °C (*D*_0_), conduction activation energy (*E*_a_^*σ*^), and ambient-temperature conductivity (*σ*_0_). These values, systematically summarized in Table 1, point out the differences in transport mechanisms across the structures considered.

**Table 1:** Activation energies for diffusion (*E*_a_^*D*^) and conduction (*E*_a_^*σ*^) processes and diffusivity (*D*_0_) and conductivity (*σ*_0_) at 25 °C of compounds considered

Sample	*E*_a_^*D*^ (eV)	*D*_0_ (cm^2^ s^−1^)	*E*_a_^*σ*^ (eV)	*σ*_0_ (S cm^−1^)	Class
NTO	0.34	1.69 × 10^−9^	0.24	3.32 × 10^−4^	Excellent (Na^+^)
NZO	0.56	6.11 × 10^−12^	0.46	1.07 × 10^−6^	Moderate (Na^+^)
bi-NTO	0.43	2.67 × 10^−11^	0.34	5.44 × 10^−6^	Moderate (Na^+^)
bi-NZO	0.48	1.66 × 10^−11^	0.38	2.84 × 10^−6^	Moderate (Na^+^)
NTZO	0.41	8.44 × 10^−11^	0.32	1.53 × 10^−5^	Moderate (Na^+^)
NZTO	0.69	2.93 × 10^−14^	0.60	5.32 × 10^−9^	Poor (Na^+^)
Na-LTNZO	0.58	4.83 × 10^−13^	0.49	4.55 × 10^−8^	Poor (Na^+^)
Li-LTNZO	0.60	8.60 × 10^−13^	0.51	8.11 × 10^−8^	Poor (Li^+^)
Na-LZNTO	0.57	8.55 × 10^−13^	0.48	7.77 × 10^−8^	Poor (Na^+^)
Li-LZNTO	0.49	9.47 × 10^−12^	0.39	8.62 × 10^−7^	Moderate (Li^+^)
LZNTO	0.51	4.66 × 10^−14^	0.42	8.48 × 10^−9^	Moderate
LTNZO	0.61	5.28 × 10^−15^	0.51	9.95 × 10^−10^	Poor

As in this section Li^+^ and Na^+^ transport properties are disclosed simultaneously, evaluation of these systems as possible dual-cation electrode is desirable. To estimate the suitability of A_2_M_6_O_13_ (A = Li, Na; M = Ti, Zr) compounds and their composites for DCS application, we establish a classification framework based on key transport parameters derived from MD simulations. Accordingly, materials having *D*_0_ ≥ 10^−9^ cm^2^ s^−1^, *E*_a_^*σ*^ ≤ 0.30 eV, and *σ*_0_ ≥ 10^−4^ S cm^−1^ are classified as “excellent” and deemed highly suitable for use as active anode materials. Compounds with moderate transport properties (*D*_0_ amounts to between 10^−12^ and 10^−9^ cm^2^ s^−1^, *E*_a_^*σ*^ between 0.30–0.45 eV, and *σ*_0_ between 10^−7^ and 10^−4^ S cm^−1^) are considered good or moderate candidates, potentially serving as either anodes or solid electrolytes depending on their structural and electrochemical context. Materials with *D*_0_ < 10^−12^ cm^2^ s^−1^, *E*_a_^*σ*^ > 0.45 eV, and *σ*_0_ < 10^−7^ S cm^−1^ are classified as poor and not recommended for direct application without further modification. These threshold values provide us with a quantitative basis to assign functional roles to each compound in dual-cation architectures.

The NZO has the largest diffusion coefficient at higher temperature and lower diffusivities for Li^+^ migration in LZNTO simple (Fig. 6a). The monocrystalline NTO and NZO samples possess distinct transport properties. NTO has excellent Na^+^ transport properties with the smallest *E*_a_^*D*^ of 0.34 eV and the largest *D*_0_ (1.69 × 10^−9^ cm^2^ s^−1^), demonstrating its superior ionic mobility, whereas NZO exhibits moderate transport behaviour (*E*_a_^*D*^ = 0.56 eV, *D*_0_ = 6.11 × 10^−12^ cm^2^ s^−1^), implying less favourable diffusion pathways. When comparing to their bi-crystalline counterparts (bi-NTO and bi-NZO), the presence of grain boundaries slightly increases *E*_a_^*D*^ affecting the Na^+^ diffusion in bi-NTO, whereas in bi-NZO the grain boundaries improve transport properties.

The bi-NTO whose parameters are *E*_a_^*D*^ = 0.43 eV, *D*_0_ = 2.67 × 10^−11^ cm^2^ s^−1^, shows a deterioration in mass transport as compared to their monocrystalline forms, whereas bi-NZO (*E*_a_^*D*^ = 0.48 eV, *D*_0_ = 1.66 × 10^−11^ cm^2^ s^−1^) enhance the migration properties, reinforcing the role of grain boundaries effect on the ionic migration. The composite materials are characterized by a more complex transport behaviour due to their multi-element composition. Both NTZO and NZTO samples have larger diffusion activation energies (0.41 and 0.69 eV, respectively) with respect to values for their pristine samples, reflecting more restricted ionic mobility as compared to simpler Na-based structures (*i.e.* NTO, NZO, bi-NTO and bi-NZO).

The conductivity at ambient temperature follows this trend, with NTZO (*σ*_0_ = 1.53 × 10^−5^ S cm^−1^) and NZTO (*σ*_0_ = 5.32 × 10^−9^ S cm^−1^) showing lower transport efficiency as compared to NTO. For Li-containing composites LZNTO and LTNZO, incorporation of Li^+^ promotes the ionic mobility in LZNTO. For instance, Li^+^ conductivity at ambient temperature in LZNTO has *E*_a_^*D*^ = 0.49 eV, *D*_0_ = 9.47 × 10^−12^ cm^2^ s^−1^, *E*_a_^*σ*^ = 0.39 eV and *σ*_0_ = 8.62 × 10^−7^ S cm^−1^, whereas in LTNZO exhibits *E*_a_^*D*^ = 0.60 eV, *D*_0_ = 8.60 × 10^−13^ cm^2^ s^−1^, *E*_a_^*σ*^ = 0.51 eV and *σ*_0_ = 8.11 × 10^−8^ S cm^−1^. However, the Na^+^ transport properties in both LZNTO and LTNZO are similar to each other. The effective transport properties (*σ*_eff_, *D*_eff_ and *E*^Δ^_a_) of both LZNTO and LTNZO composites reveals that LZNTO exhibits improved transport properties as compared with LTNZO.

The composite materials are characterized by a more complex transport behaviour due to their multi-element composition. Both NTZO and NZTO samples have larger diffusion activation energies (0.41 and 0.69 eV, respectively) with respect to values for their pristine samples. The conductivity at ambient temperature follows this trend, with NTZO (*σ*_0_ = 1.53 × 10^−5^ S cm^−1^) and NZTO (*σ*_0_ = 5.32 × 10^−9^ S cm^−1^) showing lower transport efficiency compared to NTO, reflecting more restricted ionic mobility as compared to simpler Na-based structures (*i.e.* NTO, NZO, bi-NTO and bi-NZO).

Bi-crystalline bi-NZO consistently exhibits enhanced transport performance as compared to its monocrystalline counterpart; such a result is attributed to the grain boundary-assisted diffusion, whereas grain boundaries in bi-NTO tend to impede transport.

Of all investigated samples, NTO demonstrates the most favourable transport behaviour, characterized by the lowest diffusion activation energy and the highest ionic conductivity. In composite structures containing both Zr^4+^ and Ti^4+^, the migration barriers are significantly affected, leading to elevated activation energies that restrict alkali-ion mobility.

Transport properties observed in this study align well with previous results reported in the abundant literature. For instance, diffusion coefficient values ranging from 10^−12^ to 10^−9^ cm^2^ s^−1^ were reported for well-known anode materials such as Na_2_Ti_3_O_7_, Li_4_Ti_5_O_12_ and Na_2_Ti_6_O_13_.^49–53^ Kuganathan and coworkers investigated the defect chemistry and long-range Li-ion diffusion in Li_2_Ti_6_O_13_, reporting an activation energy of 0.25 eV along the *bc*-plane using force field-based NEB computations.^27^ In our previous work, NEB calculations employing similar force field parameters yielded activation energies of 0.47 eV for Li_2_Sn_6_O_13_ and 0.52 eV for Li_2_Ti_6_O_13_.^26,28^

Notably, a significantly smaller activation energy of 0.17 eV was observed for Na^+^ migration, attributed to the presence of intermediate transition-state configurations that facilitate smoother migration pathways.^26^ In a recent study on Li-ion diffusion in mono-, bi- and composite phases of Li_2_Ti_6_O_13_ and Li_2_Zr_6_O_13_, the reported diffusion activation energies range from 0.58 to 0.66 eV.^29^ In comparison, the present work reveals a broader activation energy window of 0.34 to 0.69 eV (see Table 1).

The main distinction between the present results and previously reported NEB values lies in the computational methodology employed. MD simulations are inherently dynamic, capturing the influence of temperature, pressure, and lattice vibrations on the ion mobility. These factors enable MD to reveal thermally activated transport mechanisms and transient ionic pathways that may be inaccessible to static approaches.^23,25^ In contrast, NEB calculations based on DFT and force fields are performed at 0 K and focus exclusively on the minimum energy pathway between two fixed configurations, neglecting thermal fluctuations and dynamic lattice effects.^23,25^

Importantly, MD simulations conducted at elevated temperatures allow a direct estimation of transport properties such as diffusion coefficients and ionic conductivity under realistic thermal conditions. These temperature-dependent results are often extrapolated to ambient conditions (*e.g.*, 25 °C) using Arrhenius-type relationships, providing practical metrics for comparison with experimental data. However, such extrapolated values may differ from those derived *via* NEB computations, which reflect idealized, barrier-limited migration pathways rather than ensemble-averaged dynamics. This methodological divergence rather underscores the complementary nature of both MD and NEB approaches in elucidating ion transport phenomena and highlights the importance of contextualizing computed transport metrics within their respective theoretical frameworks.^23,25^ Despite the advantage of MD computations compared with NEB approach, experimental verification is required.

Other practical anode materials were reported.^48–50^ A combined structural and architectural modulation strategy was employed to synthesize NiMn_2_O_4_/NiCo_2_O_4_ meso-crystals *via* a solvothermal method, yielding a superlattice structure with a hollow multi-porous architecture.^54^ This engineered anode delivers a high reversible capacity of 532.2 mAh g^−1^ with 90.4% retention after 100 cycles. Such an enhanced electrochemical performance was attributed to the synergistic effects of the superlattice structure, which significantly boosts the Li^+^-ion diffusion coefficient from 2.99 × 10^−12^ to 1.19 × 10^−11^ cm^2^ s^−1^.^54^ The CoTe@Ti_3_C_2_ composite exhibits a Li^+^ diffusion coefficient of 5.20 × 10^−12^ cm^2^ s^−1^.^55^ Silicon anodes reaches Li-diffusivity of only 10^−12^ to 10^−13^ cm^2^ s^−1^, while graphite anodes reaches the value of 10^−7^ to 10^−9^ cm^2^ s^−1^.^56–58^ The predicted values collected in Table 1 align well with those values, making these compounds competitive anode candidates.^49–60^

When comparing the transport properties of LTNZO and LZNTO composites with other reported dual-cation electrodes, their performance are found to fall within the expected range. For instance, a Na-dual ion battery based on TiSe_2_-graphite exhibits Na^+^ diffusion coefficients of 3.21 × 10^−11^–1.20 × 10^−9^ cm^2^ s^−1^ and a very low diffusion barrier of 0.50 eV, leading to fast electrode kinetics akin to capacitive storage systems.^30^ In contrast, Mg^2+^/Li^+^ co-insertion into Chevrel phase Mo_6_S_8_ electrodes yields much lower diffusivities, ranging from 2.5 × 10^−16^ to 1.3 × 10^−14^ cm^2^ s^−1^ at 25 °C.^32^ More recently, quasi-1D TaS_3_ nanofibers for Mg–Li hybrid ion batteries demonstrates diffusion coefficients between 6.4 × 10^−12^ and 1.3 × 10^−10^ cm^2^ s^−1^.^31^

Against this backdrop, the dual cation composites investigated here show moderate but promising transport behaviour. LZNTO exhibits Li^+^ diffusivity of 9.47 × 10^−12^ cm^2^ s^−1^ with an activation energy of 0.49 eV and ambient conductivity of 8.62 × 10^−7^ S cm^−1^, while LTNZO yields lower diffusivity (8.60 × 10^−13^ cm^2^ s^−1^) and higher activation energy (0.60 eV), corresponding to conductivity of 8.11 × 10^−8^ S cm^−1^. Although these values are below the fastest Na-DCS, they are comparable to other hybrid electrodes such as TaS_3_ nanofibers,^31^ and significantly outperform Mg–Li Chevrel phases.^32^ These results confirm that LZNTO and LTNZO composites provide viable dual-cation transport pathways, with Li^+^ mobility compensating for Na^+^ limitations, thereby positioning them as promising anode candidates for Li–Na dual cation batteries.

**Fig. 7:**
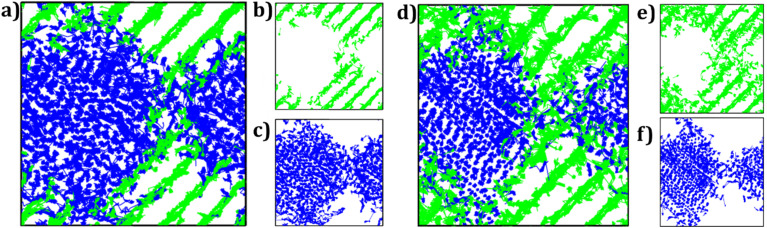
Trajectory density maps of (a) LZNTO and (d) LTNZO mixed microcrystalline samples. Blue and green lines represent the Li^+^ and Na^+^ trajectory density maps shown in panels (b), (c), (e) and (f), respectively.

Fig. 7 shows the trajectory density plots of Li-containing composites (LZNTO and LTNZO) at 900 K. The trajectory density plots of mono- and bi-crystalline samples are included in Fig. S1 (SI file). The panel 7a of Fig. 7 shows the density plot of Li^+^ and Na^+^ ions in LZNTO, while the panel 7b depicts the Na^+^ density (green lines) at Grain II and panel 7c the Li^+^ density (blue lines) at Grain I. Analogously in Fig. 7d, e and f, a higher Na^+^ density plot is observed in LTNZO as compared to that of LZNTO sample.

In addition, the A^+^-ions migrate involving an interstitial mechanism, as evidenced by density plots between [AO_4_] layers. Li^+^ ions can enter the Na^+^ sites in Grain II and Na^+^ ions migrate into the Li^+^ sites in Grain I; a similar behaviour is also observed in the LTNZO composite. Such an alkali ion exchange is beneficial for large-scale alkali migration, enhancing their overall transport properties. This kind of mixed alkali materials was reported before.^36,61–63^ For instance, metal–sulphur (Li/Na–S) battery technology was considered to be one of the most promising battery systems owing to its high specific capacity.^62^ A combination of Fe-based metal organic framework with Li/Na–S was reported taking the Fe-based metal organic framework as cathode resulting in a combined Li/Na–S battery.^63^ Sodium titanate nanowire was evaluated as an anode for dual Li/Na ion batteries.^54^ The Na^+^ storage was found to be more efficient than the Li^+^ counterpart without relevant phase changes during the cycling, thus avoiding the capacity fade.^36^

### 3.3 Possible experimental realization of predicted composite compounds

The transport properties predicted above for the composite compounds deserve to be considered for practical applications, and some possible experimental syntheses of these promising composites can be designed.^59–64^

The most common synthetic routes are solid-state reactions, ion-exchange and Pechini (sol–gel) methods.^7,8,21,22,59–63^ For instance, a solid-state synthesis of the Li_2_Ti_6_O_13_–Na_2_Zr_6_O_13_ biphasic compound (LTNZO or LZNTO) could be achieved by thoroughly mixing stoichiometric amounts of high-purity precursors including Li_2_CO_3,_ TiO_2_, Na_2_CO_3_, and ZrO_2_.

A more controlled method involves the alkali ion-exchange, starting from pure Na_2_Ti_6_O_13_.^7,9,10^ Na_2_Ti_6_O_13_ can undergo partial lithium exchange when treated with molten LiNO_3_ at high temperature (>300 °C).^7,8^ By carefully controlling the duration and temperature of the exchange process, it is then possible to achieve a partial substitution of Na^+^ by Li^+^, resulting in a stable mixture of Li_2_Ti_6_O_13_ and Na_2_Ti_6_O_13_.^7,8^ This method is particularly attractive because it tends to preserve the tunnel structure of the titanate framework and allows fine-tuning of the Li^+^/Na^+^ ratio, which can be critical for tailoring electrochemical or transport properties.^7,8,10^ There is no report yet regarding an experimental route for Na_2_Zr_6_O_13_ and Li_2_Zr_6_O_13_, but theoretically a Zr^4+^/Ti^4+^ ion-exchange can be achieved generating high thermodynamically stable compounds.^13,65^

A third option involves the Pechini method^65,66^ which offers a superior control over chemical homogeneity and particle morphology. In this approach, metal precursors such as LiNO_3_, NaNO_3_, and a titanium source (*e.g.* TiCl_4_) can be used and by adjusting the Li^+^/Na^+^ ratio in the precursors the relative amounts of Li_2_Ti_6_O_13_ and Na_2_Ti_6_O_13_ in the desirable product could be influenced.^65,66^

In addition, a possible sol–gel synthesis route for obtaining a Li_2_Zr_6_O_13_–Na_2_Ti_6_O_13_ (NZTO) compound involves using zirconium(iv) propoxide or zirconium oxychloride as the zirconium source, combined with sodium nitrate (or acetate) and alkali precursors. Although this method is more complex than a solid-state synthesis, it enables better control over microstructure and phase distribution which can be advantageous for applications requiring fine-tuned material properties. Similar sol–gel routes can be proposed to obtain LTNZO and LZNTO compounds.

Given the promising transport properties predicted in this study, it is highly desirable to further explore synthetic routes, particularly the sol–gel and ion-exchange methods, for the controlled fabrication of biphasic compounds. These materials exhibit strong characteristics that position them as potential dual-function anode candidates for both lithium- and sodium-ion batteries.

## 4 Concluding remarks

Molecular dynamics simulations conducted in the present study provide us with insights into transport properties of Na_2_Ti_6_O_13_ (NTO) and Na_2_Zr_6_O_13_ (NZO) as effective anode materials for alkali-ion batteries. The findings reveal that transport properties of bi-crystalline samples (bi-NTO, bi-NZO) are enhanced with respect to their monocrystalline counterparts, due to the grain boundary-assisted diffusion. The bi-NZO turns out to get an improved Na^+^ migration whereas the bi-NTO experiences a slight deterioration in transport properties.

Li-containing composites (LZNTO, LTNZO) result in superior conductivity as compared to Na-based composites (NTZO, NZTO), reinforcing the higher mobility of Li^+^ ions within solid-state materials. This study provides evidence that LZNTO and LTNZO composites can function as dual cation anodes, combining Li^+^ mobility with Na^+^ abundance to enable simultaneous transport pathways for advanced alkali ion batteries. Of all samples studied, NTO is determined to have the most favourable transport properties, possessing the smallest diffusion activation energy and the highest conductivity, highlighting its efficient anode material.

Furthermore, incorporation of both Zr^4+^ and Ti^4+^ ions in composite structures leads to increased activation energies that restrict the alkali ion transport, thereby impacting the overall efficiency of charge transfer. In contrast, the presence of mixed alkali elements (Li^+^ and Na^+^ composites) tends to facilitate interstitial ion migration, thereby enable efficient ion exchange mechanisms that finally contribute to improved large-scale alkali ion transport. These results again underscore the importance of structural modifications and elemental composition in optimizations of ionic conductivity, providing us with valuable guidance for the design and selection of advanced anode materials for battery applications.

## Conflicts of interest

There are no conflicts to declare.

## Supplementary Material

RA-016-D5RA10064J-s001

## Data Availability

The data supporting this article have been included as part of the supplementary information (SI). Supplementary information: Table S1 with cation, vacancy, and defect counts for various A_2_M_6_O_13_ samples, example LAMMPS input files for NTO, bi-NTO, and LTNZO systems to ensure reproducibility, and Fig. S1 showing Na^+^ trajectory density maps that illustrate ion transport pathways. See DOI: https://doi.org/10.1039/d5ra10064j.
