# Supplementary material for: Composite A2M6O13 anodes (A = Li, Na; M = Ti, Zr) for Li–Na dual cation batteries: a theoretical investigation
Source: RSC Adv. 2026 Mar 2;16(13):11908–17. doi: 10.1039/d5ra10064j (PMC12951593; doi:10.1039/d5ra10064j)
Supplement: RA-016-D5RA10064J-s001 [file RA-016-D5RA10064J-s001.pdf]

Supplementary Information (SI) for RSC Advances.
This journal is © The Royal Society of Chemistry 2026

Electronic Supplementary Information

Composite $A_2M_6O_{13}$ Anodes (A = Li, Na; M = Ti, Zr) for Li-Na Dual Cation Batteries: A Theoretical Investigation

Duc Toan Truong,[a,b] Yohandys A. Zulueta,[c,*] My Phuong Pham-Ho,[d,e] An Giang Nguyen,[f] Chi M. Phan[g] and Minh Tho Nguyen[f,*]

[a] *Laboratory for Chemical Computation and Modeling, Institute for Computational Science and Artificial Intelligence, Van Lang University, Ho Chi Minh City, Vietnam*
[b] *Faculty of Applied Technology, Van Lang School of Technology, Van Lang University, Ho Chi Minh City, Vietnam.*
[c] *Departamento de Física, Facultad de Ciencias Naturales y Exactas, Universidad de Oriente, CP 90500, Santiago de Cuba, Cuba. Email: yzulueta@uo.edu.cu*
[d] *Faculty of Chemical Engineering, Ho Chi Minh City University of Technology (HCMUT), 268 Ly Thuong Kiet Street, Ho Chi Minh City, Viet Nam*
[e] *Vietnam National University Ho Chi Minh City, Linh Trung, Thu Duc, Ho Chi Minh City, Viet Nam*
[f] *Center for Environmental Intelligence, VinUniversity, Gia Lam, Hanoi, Vietnam. Email: tho.nm@vinuni.edu.vn*
[g] *College of Engineering and Computer Science, VinUniversity, Gia Lam, Hanoi, Vietnam.*
[h] *Discipline of Chemical Engineering, WASM MECE, Curtin University, Perth, WA 6045 Australia*

Table S1: Number of ionic species in each sample, including cations, vacancies, and defect concentrations.

Sample	N_{Na}^{+}	N_{Li}^{+}	N_{Ti}^{4+}	N_{Zr}^{4+}	N_{O}^{2-}	Vac-O^{2-}	Vac-A^{+}	A^{+} defect concentration
**NTO**	1400		4320		9340	40	20	0.0927
**NZO**	1400			4320	9340	40	20	0.0927
**bi-NTO**	1546		4762		10297		34	0.0931
**bi-NZO**	1546			4762	10297		34	0.0931
**NTZO**	1546		2386	2376	10297		34	0.0931
**NZTO**	1546		2376	2386	10297		34	0.0931
**LTNZO**	774	772	2376	2386	10297		34	0.0931
**LZNTO**	774	772	2386	2376	10297		34	0.0931

Examples of LAMMPS input files (at 900K) of NTO, bi-NTO samples, and LTNZO composite are included for reproducibility providing further simulation details for the readers. A zip file is included with the simulation boxes.

1. Input file of NTO

```
###########################################################
##
#------------Variables and cell-------------------------#
###########################################################
##

clear
```

```
units        metal       #eV,atomic charge,angstroms,ps,kelvin,bars,g/mol
dimension 3
boundary p p p
atom_style charge
#processors * * * grid numa
read_data 4x15x6NeutralNTO.lmp

group          Na type 1
group          Ti type 2
group          O  type 3
#group                 Zr type 4


mass 1   23                #Na
mass 2   47.867          # Ti
mass 3   15.99900000         # O

variable T1 equal 900
variable Timer equal step*dt
log ${T1}.lammps

##########################################################
#
#---------- Pair styles and electrostatics--------------#
##########################################################
#

pair_style buck/coul/long 10
pair_coeff * *  0.0 1.0 0.0
pair_coeff 1 1  0.0 1.0 0.0
pair_coeff 1 2  0.0 1.0 0.0
pair_coeff 1 3  1271.504 0.3 0.0 #Na-O
pair_coeff 2 2  0.0 1.0 0.0
pair_coeff 2 3 5111.7  0.2625   0.0 #Ti-O
pair_coeff 3 3  22764.3 0.149 27.627 #O-O



kspace_style pppm 1e-05

##########################################################
# -------------- Run Minimization ---------------------#
##########################################################

reset_timestep 0
```

```
timestep 0.5
thermo 10
thermo_style custom step enthalpy fmax lx ly lz vol press
min_style cg
minimize 1e-25 1e-25 5000 10000


#######################################################
# -------------- Relax Cell ---------------------------#
#######################################################

reset_timestep 0
timestep 0.5
fix 1 all box/relax aniso 1.0 vmax 0.003
thermo 1
thermo_style custom step enthalpy fmax lx ly lz vol press
min_style cg
minimize 1e-25 1e-25 5000 10000
unfix 1

#######################################################
# ------------- Run NPT T1----------------------------#
#######################################################

reset_timestep 0
timestep 0.002
velocity all create ${T1} 49284 rot yes dist gaussian
fix     2 all npt temp ${T1} ${T1} 0.01 aniso 1.0 1.0 0.1
thermo_style custom step v_Timer cpu temp etotal fmax lx ly lz vol press
thermo 1000
run 5000
unfix 2

reset_timestep 0
timestep 0.002

compute        mymsdNa Na msd com yes
compute        mymsdO O msd com yes
variable       msdxNa equal "c_mymsdNa[1]"
variable       msdyNa equal "c_mymsdNa[2]"
variable       msdzNa equal "c_mymsdNa[3]"
variable       msdtotNa equal "c_mymsdNa[4]"
variable       msdoxO equal "c_mymsdO[1]"
variable       msdoyO equal "c_mymsdO[2]"
variable       msdozO equal "c_mymsdO[3]"
```

```
variable       msdototO equal "c_mymsdO[4]"

fix           msdT2 O ave/time 1 1 2500 v_msdoxO v_msdoyO v_msdozO v_msdototO
file msdOxygen${T1}
fix          msdT1 Na ave/time 1 1 2500 v_msdxNa v_msdyNa v_msdzNa v_msdtotNa
file msdSodium${T1}
fix           3 all nvt temp ${T1} ${T1} 0.01
thermo_style custom step v_Timer cpu temp etotal fmax lx ly lz vol press
v_msdtotNa v_msdototO
thermo 5000
dump dynamics all xyz 5000 monoNTO_${T1}.xyz
dump_modify dynamics every 5000 element Na Ti  O  first yes
run          1000000
unfix 3
unfix msdT1
unfix msdT2
```

2. Input file of bi-crystalline bi-NTO

```
##########################################################
##
#------------Variables and cell-------------------------#
##########################################################
##

clear
units         metal        #eV,atomic charge,angstroms,ps,kelvin,bars,g/mol
dimension 3
boundary p p p
atom_style charge
#processors * * * grid numa
read_data NeutralNTO-1.lmp

group          Na type 1
group          Ti type 2
group          O  type 3
#group                 Zr type 4


mass 1   23                 #Na
mass 2   47.867           # Ti
mass 3   15.999           # O

variable T1 equal 900
variable Timer equal step*dt
```

```
log ${T1}.lammps

##############################################################
#
#---------- Pair styles and electrostatics--------------#
##############################################################
#

pair_style buck/coul/long 10
pair_coeff * *  0.0 1.0 0.0
pair_coeff 1 1  0.0 1.0 0.0
pair_coeff 1 2  0.0 1.0 0.0
pair_coeff 1 3  1271.504 0.3 0.0 #Na-O
pair_coeff 2 2  0.0 1.0 0.0
pair_coeff 2 3 5111.7  0.2625   0.0 #Ti-O
pair_coeff 3 3  22764.3 0.149 27.627 #O-O
kspace_style pppm 1e-05

##############################################################
# -------------- Run Minimization ---------------------#
##############################################################

reset_timestep 0
timestep 0.5
thermo 10
thermo_style custom step enthalpy fmax lx ly lz vol press
min_style cg
minimize 1e-25 1e-25 5000 10000


##############################################################
# -------------- Relax Cell ---------------------------#
##############################################################

reset_timestep 0
timestep 0.5
fix 1 all box/relax aniso 1.0 vmax 0.003
thermo 1
thermo_style custom step enthalpy fmax lx ly lz vol press
min_style cg
minimize 1e-25 1e-25 5000 10000
unfix 1

##############################################################
# ------------- Run NPT T1-----------------------------#
```

```
##########################################################

reset_timestep 0
timestep 0.002
velocity all create ${T1} 49284 rot yes dist gaussian
fix     2 all npt temp ${T1} ${T1} 0.01 aniso 1.0 1.0 0.1
thermo_style custom step v_Timer cpu temp etotal fmax lx ly lz vol press
thermo 1000
run 5000
unfix 2

reset_timestep 0
timestep 0.002

compute       mymsdNa Na msd com yes
compute       mymsdO O msd com yes
variable      msdxNa equal "c_mymsdNa[1]"
variable      msdyNa equal "c_mymsdNa[2]"
variable      msdzNa equal "c_mymsdNa[3]"
variable      msdtotNa equal "c_mymsdNa[4]"
variable      msdoxO equal "c_mymsdO[1]"
variable      msdoyO equal "c_mymsdO[2]"
variable      msdozO equal "c_mymsdO[3]"
variable      msdototO equal "c_mymsdO[4]"

fix          msdT2 O ave/time 1 1 2500 v_msdoxO v_msdoyO v_msdozO v_msdototO
file msdOxygen${T1}
fix         msdT1 Na ave/time 1 1 2500 v_msdxNa v_msdyNa v_msdzNa v_msdtotNa
file msdSodium${T1}
fix          3 all nvt temp ${T1} ${T1} 0.01
thermo_style custom step v_Timer cpu temp etotal fmax lx ly lz vol press
v_msdtotNa v_msdototO
thermo 5000
dump dynamics all xyz 5000 2grainsNTO_${T1}.xyz
dump_modify dynamics every 5000 element Na Ti  O  first yes
run          1000000
unfix 3
unfix msdT1
unfix msdT2
```

3. Input file of LTNZO composite

```
##########################################################
##
#------------Variables and cell-------------------------#
```

```
################################################################
##

clear
units          metal        #eV,atomic charge,angstroms,ps,kelvin,bars,g/mol
dimension 3
boundary p p p
atom_style charge
#processors * * * grid numa
read_data LZNTO-1.lmp

group          Na type 1
group         Li type 2
group          Ti type 3
group          O  type 4
group          Zr type 5


mass 1   23           # Na
mass 2   6.94          # Li
mass 3   47.867        # Ti
mass 4   15.999        # O
mass 5   91.224        # Zr



set              group O charge -2.0
set              group Li charge 1.0
set              group Ti charge 4.0
set              group Zr charge 4.0
set              group Na charge 1.0

variable T1 equal 1000
variable Timer equal step*dt
log ${T1}.lammps

################################################################
#
#---------- Pair styles and electrostatics--------------#
################################################################
#

pair_style buck/coul/long 10
pair_coeff * *  0.0 1.0 0.0
pair_coeff 1 4  1271.504 0.3 0.0 #Na-O
```

```
pair_coeff 2 4  632.1018 0.2906  0.0 #Li-O
pair_coeff 3 4 5111.7  0.2625   0.0 #Ti-O
pair_coeff 4 5 985.87 0.3760  0.0 #Zr-O
pair_coeff 4 4  22764.3 0.149 27.627 #O-O



kspace_style pppm 1e-05

#######################################################
# -------------- Run Minimization ---------------------#
#######################################################

reset_timestep 0
timestep 0.5
thermo 10
thermo_style custom step enthalpy fmax lx ly lz vol press
min_style cg
minimize 1e-25 1e-25 5000 10000


#######################################################
# -------------- Relax Cell ---------------------------#
#######################################################

reset_timestep 0
timestep 0.5
fix 1 all box/relax aniso 1.0 vmax 0.003
thermo 1
thermo_style custom step enthalpy fmax lx ly lz vol press
min_style cg
minimize 1e-25 1e-25 5000 10000
unfix 1

#######################################################
# ------------- Run NPT T1----------------------------#
#######################################################

reset_timestep 0
timestep 0.002
velocity all create ${T1} 4928 rot yes dist gaussian
fix      2 all npt temp ${T1} ${T1} 0.01 aniso 1.0 1.0 0.1
thermo_style custom step v_Timer cpu temp etotal fmax lx ly lz vol press
thermo 1000
```

```
run 5000
unfix 2

reset_timestep 0
timestep 0.002
compute       mymsdNa Na msd com yes
variable      msdx equal "c_mymsdNa[1]"
variable      msdy equal "c_mymsdNa[2]"
variable      msdz equal "c_mymsdNa[3]"
variable      msdtotNa equal "c_mymsdNa[4]"

compute       mymsdLi Li msd com yes
variable      msdx equal "c_mymsdLi[1]"
variable      msdy equal "c_mymsdLi[2]"
variable      msdz equal "c_mymsdLi[3]"
variable      msdtotLi equal "c_mymsdLi[4]"

fix           msdT1 Li ave/time 1 1 5000 v_msdx v_msdy v_msdz v_msdtotLi file
MSDLi${T1}
fix           msdT2 Na ave/time 1 1 5000 v_msdx v_msdy v_msdz v_msdtotNa file
MSDNa${T1}
fix         3 all nvt temp ${T1} ${T1} 0.01
thermo_style custom step v_Timer cpu temp etotal fmax lx ly lz vol press
v_msdtotLi v_msdtotNa
thermo 5000
dump dynamics all xyz 5000 2grainsLZNTO_${T1}.xyz
dump_modify dynamics every 5000 element Li Na Ti O Zr  first yes
run         1000000
unfix 3
unfix msdT1
unfix msdT2
```

Trajectory density maps

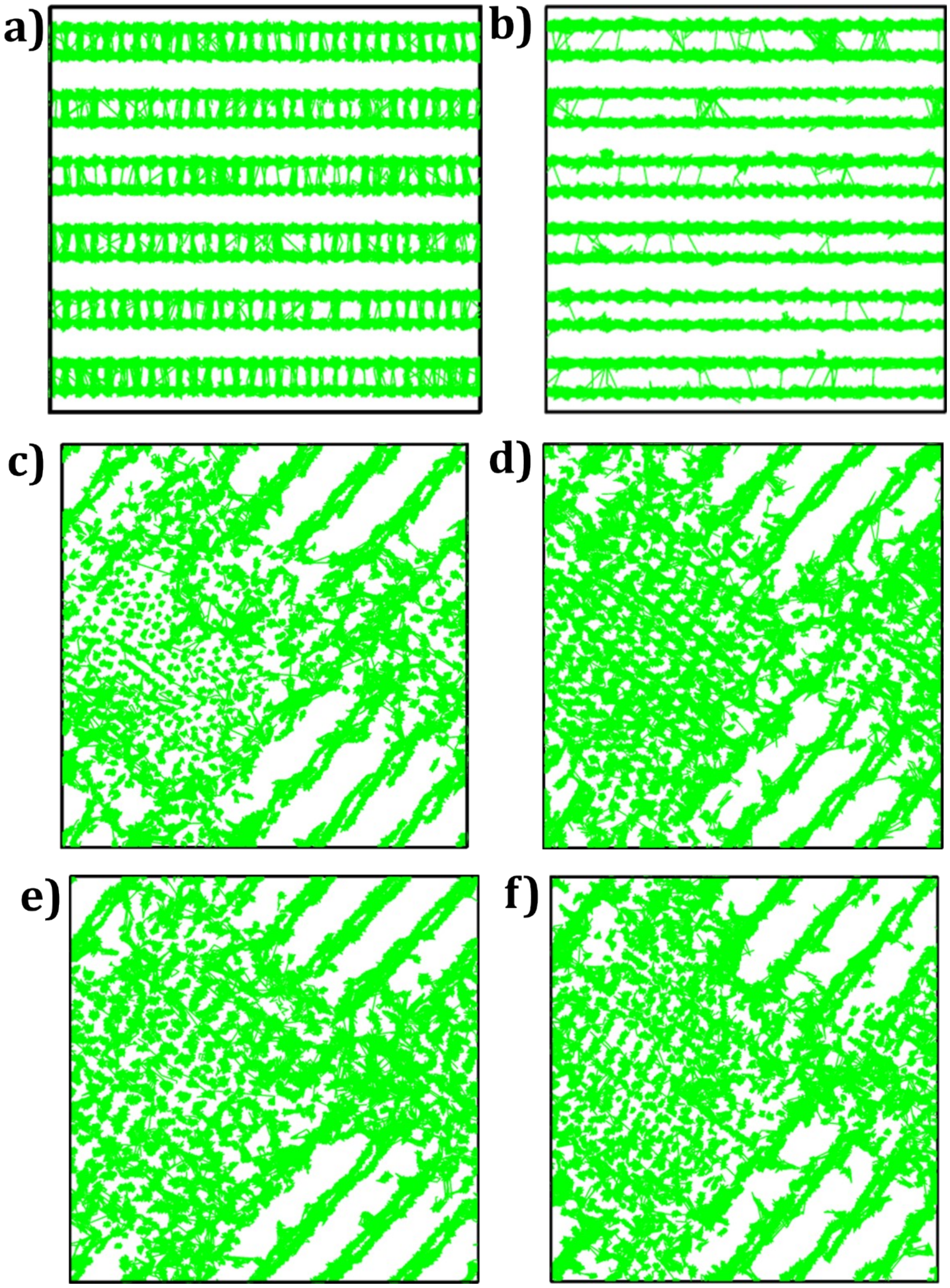

Figure S1: Na^+ trajectory density maps (green lines) of: a) NTO and b) NZO, c) bi-NTO, d) bi-NZO, e) NTZO and f) NZTO samples.
